# Training community hospital residents for a future in the hospital's community – what program directors should know about Stark Laws

**DOI:** 10.3402/jchimp.v3i2.20735

**Published:** 2013-07-05

**Authors:** Richard Alweis

**Affiliations:** 1 Department of Medicine, Jefferson Medical College, Philadelphia, PA, USA; 2 Department of Medicine, Reading Health System, West Reading, PA, USA

Some have argued that physicians have difficulty regulating themselves (an argument recently echoed by the ACGME, in fact), and they often point to physician laboratory and radiology self-referral abuses that occurred throughout the 1980s as their major talking point. These discussions led to a series of federal laws, referred to collectively as the Stark Laws (after California Congressman Pete Stark who introduced the legislation), that regulated how physicians interact with ancillary services with regard to patients that have Medicaid or Medicare (initially, though this has since been expanded) (1, 2). While the majority of program directors likely have in-house counsel to assist them/manage all Stark-related issues, it behooves program directors to have a basic working knowledge of the laws to appropriately counsel graduating residents regarding postresidency employment agreements and maintain professionalism standards.

The Stark Laws were rolled out in three phases. Stark 1 became effective January 1, 1992, and governed self-referral for laboratory services in Medicare patients. Stark 2 began to be enforced on July 26, 2004 (though the provisions for the law were already written and went into effect on January 1, 1995), and extended the prohibitions for self-referral to Medicaid patients, governed more ancillaries, including radiology, and had direct effects on physician recruitment, incentive plans, rental of space and office equipment, risk-sharing arrangement, and other necessities of daily practice life. Stark 3 became effective December 4, 2007, and extended Stark rules to physicians that do not accept Medicare and Medicaid reimbursements and rearranged official definitions for fair market value, ‘incident to’ services, and ‘physicians in a group’ (3). In a reversal, Stark 3 removed the safe harbor for fair market values established in Stark 2. It also indicated that a member of a group of physicians stands ‘in the shoes of’ his/her entire group when considering relationships involved in billing for designated health services (DHS). These DHS include: clinical laboratory services, physical therapy services, occupational therapy services, radiology services (MRI, CT, ultrasound, and nuclear medicine), radiation therapy services and supplies, durable medical equipment (DME) and supplies, prosthetics and prosthetic supplies, home health services, outpatient prescription services, and inpatient and outpatient hospitalization services (1).

Therefore, the direct application of the Stark Laws to most internal medicine residency programs is presently somewhat limited in scope. However, radiation oncology residency programs have run afoul of Stark legislation in the recent past, and, with more physicians employed in hospitals than previously, it may come to pass that our practices will be more tightly examined. Program directors are responsible for upholding the professionalism of their residents and of their own programmatic planning, and must pay close attention to any practice that will result in significant financial liability. Relying solely on a legal department to advise a program and having no basic understanding of the law, especially in smaller hospitals that may not have in-house counsel, puts a program director and resident at unnecessary risk. Some common scenarios are explored below.

If you want to hire your graduating resident into your hospital staff and give tuition reimbursement, relocation expenses, or partial support (for example, funding the roll-out of an electronic medical record at their office), this is likely allowable at the present time. Since most residents are not considered full members of the medical staff while residents, and since most residents are not being induced to move a practice into the geographic region that a hospital serves (i.e., receiving large financial incentives) given they are already practicing there, the resident likely falls within all of the exceptions under §411.357(e) of the Stark code (1, 2). However, the government has been closing the exceptions to the Stark Laws, so each legislative session will bring new complexity and potential risk. At present, allowable relationships with residents do include full employment by the institution after graduation, with the assumptions that:

1. the salary offered represents fair market value;
2. the salary is given for identifiable services rendered;
3. the employment contract is for at least 1 year; and
4. there is no requirement for remuneration to be directly linked to the volume of services generated, with the exception to the exception of a physician incentive plan (2, 3).

The physician incentive plan exception to the exception requires that a payment cannot be made to a physician or a group to limit their provision of medically necessary services to a patient of the organization (i.e., one cannot pay a physician to NOT perform certain tests in the office, for example, electrocardiogram). A further caveat is that any significant downstream financial risks from the plan to the physician meet another set of strict regulations (§422.208) (1, 2).

If your institution wants to help to partially finance a start-up for the resident, but the resident is not technically employed by the hospital, it is a more complicated situation. The resident must be allowed to establish staff privileges at any hospital they choose to and to refer business wherever they want. The resident's practice must be within the ‘geographic area served by the hospital’, which is defined by the ‘75% rule’. This rule defines the geographic area served by a hospital as the area composed of the lowest number of contiguous zip codes from which the hospital draws at least 75% of its inpatients. There are significant regulations around movement of practices, but this discussion is limited to our residents when they graduate from residency.

The penalties for Stark Law violations are generally quite severe, and include refunds of payments, denial of payments for services rendered, and a significant punitive damage ($100,000 per each part of an agreement considered to be an attempt to circumvent the Stark Laws) (1). How Accountable Care Organizations (ACOs) will finally meld with the existing Stark Laws is also still evolving, and exceptions from the Stark Law regulations (or facets of) are often sought by ACOs (and granted by the Federal Trade Commission). The Affordable Care Act does provide for leniency on the damages for institutions that voluntarily disclose infractions between hospitals and health care providers. As program directors are often the first person a resident will seek advice from when in contract negotiations, this further raises the stakes, as your discovery of an infraction, and reporting to the appropriate legal authority, may save the organization from severe financial damages. In the end, however, even basic working knowledge of Stark Laws remains limited, and it behooves program directors to foster a good working relationship with their hospital legal division to provide appropriate counseling to residents and maintain their and our professionalism.
